# Use of Technology to Support Health Care Providers Delivering Care in Low- and Lower-Middle-Income Countries: Systematic Umbrella Review

**DOI:** 10.2196/66288

**Published:** 2025-06-18

**Authors:** Adam Craig, Harriet Lawford, Maggie Miller, Liuyi Chen-Cao, Leanna Woods, Siaw-Teng Liaw, Myron Anthony Godinho

**Affiliations:** 1 Operational Research and Decision Support for Infectious Diseases Program Centre for Clinical Research The University of Queensland Herston, QLD Australia; 2 National Centre for Epidemiology and Population Health The Australian National University Canberra Australia; 3 Queensland Digital Health Centre The University of Queensland Herston Australia; 4 School of Clinical Medicine and School of Population Health UNSW Sydney Randwick Australia; 5 Westmead Applied Research Centre The University of Sydney Sydney Australia

**Keywords:** digital health, eHealth, Sustainable Development Goals, primary health care, universal health coverage, development

## Abstract

**Background:**

Health care providers are at the forefront of the digital health transformation underway in low- and lower-middle-income countries (LLMICs). Digital health innovations (DHIs) promise more efficient and equitable health care delivery. However, their implementation often outpaces the generation of evidence supporting their effectiveness, resulting in fragmented projects that are poorly aligned with local system needs. Recognizing the diverse ways DHIs are used, the World Health Organization introduced a revised *Classification of Digital Health Interventions* in 2023 identifying 4 primary user groups, including health care providers.

**Objective:**

This study aims to synthesize the current evidence on the use and impact of DHIs by health care providers in LLMICs.

**Methods:**

We conducted an umbrella review of articles published between 2010 and 2024. Articles were sourced through PubMed, Embase, Scopus, and Web of Science. The search strategy combined keywords with Boolean operators. To be included, articles had to be original research published as systematic, thematic, or scoping reviews and had to use a systematic process for data identification and extraction. They needed to relate to a DHI implemented in at least 1 LLMIC and be available in English. World Bank country classifications were used to define LLMICs. Data extracted were deductively coded and thematically analyzed according to the 11 health system functions outlined in the 2023 World Health Organization classification of DHIs.

**Results:**

Overall, 88 reviews were included. Telemedicine was the most commonly studied DHI (60/88, 68%), with evidence suggesting that it has improved information sharing among providers (eg, hospitals and private providers) and enhanced delivery efficiency, particularly in limited-access settings. Outside of telemedicine, the evidence remains thin and uneven across other categories of DHIs. While DHIs appear to help providers interact more effectively with clients, systems, and one another, many interventions remain short lived, limited in scale, or contextually misaligned. The use of personal mobile devices by health care providers emerged as a common and practical platform for delivering DHIs, highlighting the potential for cost savings and rapid uptake. Persistent challenges such as insufficient infrastructure, high setup costs, and limited workforce capacity remain key barriers to sustainable scale-up.

**Conclusions:**

Some evidence suggests DHIs are transforming health care delivery in LLMICs and contributing to broader health goals; however, robust and conclusive evidence on DHIs’ impact on health outcomes, cost-effectiveness, and long-term sustainability is lacking. Caution is warranted when introducing DHIs that may not align with underlying system constraints. Policy makers and development partners are encouraged to support implementation research to build a more coherent global evidence base. DHIs should be seen not as a stand-alone solution but as a complementary tool to strengthen health systems.

**Trial Registration:**

PROSPERO CRD42024586285; www.crd.york.ac.uk/PROSPERO/view/CRD42024586285

## Introduction

The digital revolution has ushered in a new era of possibilities for health care systems worldwide, including electronic health records, telemedicine, artificial intelligence, and remote patient monitoring. In the evolving health care ecosystem, health care providers have emerged at the forefront of this transformation, leveraging digital technologies to navigate and tackle the multifaceted challenges of delivering care when and where it is needed, particularly in settings with constrained resources, fragmented systems, and limited access to essential services.

The adoption of digital technology in health care has been fueled by several factors: cost-effectiveness; the demand for data-driven decision-making; technological advancements; and new analytic methods, such as machine learning. These developments have created opportunities to gather, process, exchange, and analyze medical and health service data securely and in near real time, aiding the delivery of data-driven clinical care and service management in both resource-poor and resource-rich countries. For low- and lower-middle-income countries (LLMICs), these promises are compelling; however, real-world implementation often occurs in the absence of foundational infrastructure, adequate digital literacy among users, or clear governance frameworks.

Governments, donors, and multilateral institutions recognize the opportunities that digital technologies present. In May 2018, countries unanimously adopted a World Health Assembly resolution calling for the World Health Organization (WHO) to develop a digital strategy to support universal health coverage (UHC) and ultimately help countries achieve the health aims of the Sustainable Development Goals (SDGs) [1,2].

Amid the heightened interest, digital health innovations (DHIs) have often been rolled out without thorough evaluation, resulting in a proliferation of short-lived projects and diverse digital tools [3,4]. Consequently, there is limited consensus on the impact that digital interventions may have on health systems and health outcomes at scale [5-7]. This concern was highlighted by the WHO Bellagio eHealth Evaluation Group, which stated the following: “To improve health and reduce health inequalities, rigorous evaluation of eHealth is necessary to generate evidence and promote the appropriate integration and use of technologies” [8].

While recognizing the innovative role digital technologies can play in strengthening health systems, a better understanding of their contribution is needed to support future investment decisions. This is particularly important in LLMIC contexts, where health service delivery is resource sensitive and where digital health must provide a demonstrable benefit to justify the allocation of resources that could otherwise be spent on facilities, equipment, staff, medicines, and other essential commodities.

To guide and harmonize digital health development, the WHO released the *Classification of Digital Health Interventions, Services, and Applications in Health* (*CDISAH*) in 2023 [9]. The framework categorizes DHIs into four groups based on their primary users: (1) persons (ie, consumers of health care services), (2) health care providers, (3) health care managers and support personnel, and (4) data service managers.

This umbrella review explores the evidence for the effective use of DHIs by health care providers (the second DHI user group of the *CDISAH*) in LLMICs to address health system challenges. This paper is the first in a planned series synthesizing evidence on DHI use across all 4 user groups. It focuses on health care providers, given the concentration of existing literature on DHIs designed for them and their central role in delivering frontline services critical to achieving UHC. Here, we synthesize evidence about how, to date, DHIs have been used to support health service delivery in LLMICs and the extent to which they help overcome enduring health system constraints. This review is registered at PROSPERO (CRD42024586285).

## Methods

### Overview

Given the plethora of published literature on DHIs, an umbrella review was conducted. An umbrella review is a “systematic collection and assessment of multiple reviews done on a specific research topic” [10]. We followed the process developed by Arksey and O’Malley [11], which involved five steps: (1) identification of research questions; (2) identification of relevant studies; (3) study screening and selection; (4) charting the data; and (5) collating, summarizing, synthesizing, and reporting results. The review was conducted in accordance with the PRISMA (Preferred Reporting Items for Systematic Reviews and Meta-Analyses) guidelines (Multimedia Appendix 1). A protocol for this study is published elsewhere [12].

### Step 1: Research Questions

Two research questions were developed: (1) How are health care providers in LLMIC using DHIs to address health system challenges? and (2) To what extent are DHIs helping to overcome health system constraints? A health care provider is defined as a member of the health workforce who delivers health services, including doctors, nurses, community-based health care providers, allied health professionals, and volunteers who support the provision of health care. DHIs are defined as digital and mobile technologies used to support health system needs [2].

### Step 2: Identifying Studies

A predefined search strategy encompassing keywords and medical subject headings covering 3 concepts—digital health, UHC, and LLMICs—was used. We searched 4 electronic databases—PubMed, Embase, Scopus, and Web of Science—in April 2023 and again in August 2024. The search strategy used a combination of keywords with Boolean operators in the following syntax: [“Digital Health” (and related terms)] AND [“Universal Health Coverage” (and related terms)] AND [“LLMICs” (and related terms)]. Medical subject headings and Emtree terms were used where appropriate. Limiters applied included “humans,” “English,” “published between January 2010 and August 2024 (approximately 15 years),” and “review articles only.” Full search terms can be found in Multimedia Appendix 2.

### Step 3: Study Screening and Selection

The eligibility criteria for inclusion were original research publications with the following characteristics. To be included, publications needed to be review articles that (1) described a systematic data identification and extraction method, (2) focused on a DHI implemented in at least 1 LLMIC, (3) were available in English, and (4) were published between January 2010 and August 2024. Studies were excluded if they were not reviews or did not report on DHIs implemented in at least 1 LLMIC. The 2024-2025 World Bank country income classifications were used to define LLMICs [13]. Studies were filtered using the software Covidence (Veritas Health Innovation Ltd), with duplicates removed. Two reviewers (AC and MAG) independently screened titles and abstracts against the predefined inclusion and exclusion criteria. Discrepancies or uncertainties were resolved through discussion. In cases of disagreement or ambiguity regarding eligibility, a conservative approach was adopted, and the manuscript was retained for full-text review. Full-text review was conducted by AC and MAG.

### Step 4: Charting the Data

In the first stage of data extraction, the following information was collected in an Excel (Microsoft) spreadsheet: citation details, World Bank income classification level, study location defined by WHO region, evidence type (qualitative, quantitative, or both), research methods, the health conditions or applications the DHI addressed, synthesis of evidence, and reflection on the contribution and success or failures of the DHI.

In the second stage, extracted information was coded according to the 11 DHIs designed for health care providers, as per the *CDISAH*. These are DHIs used by health care providers to (1) identify and register persons, (2) generate a person-centered health record, (3) provide decision support, (4) support telemedicine, (5) enable communication among health care providers, (6) facilitate referral coordination, (7) support scheduling and activity planning, (8) deliver training, (9) manage prescription and medication, (10) manage laboratory and diagnostic imaging, and (11) conduct financial transactions [9].

### Step 5: Collating, Summarizing, and Reporting the Results

We performed a content and thematic analysis of data extracted from the included papers and presented them by the 11 categories mentioned earlier.

### Ethical Considerations

Ethical clearance for this study was not required.

## Results

### Overview

**Figure 1:**
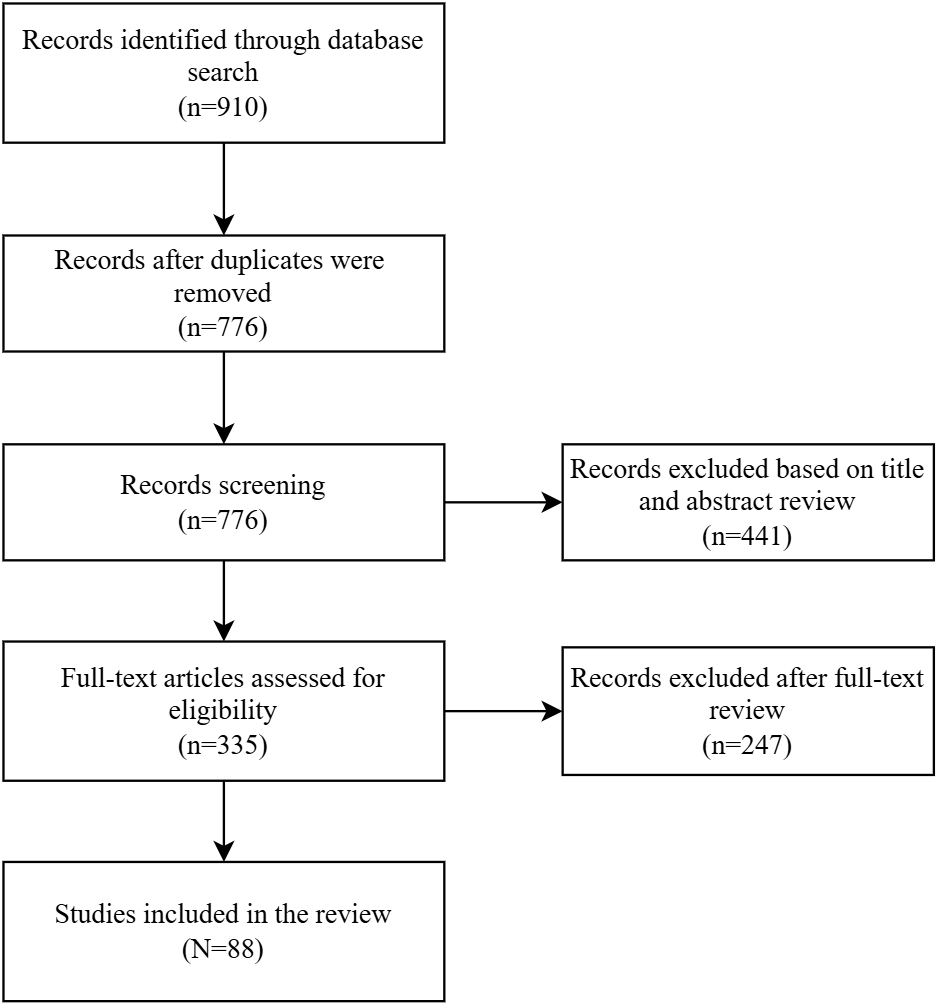
PRISMA (Preferred Reporting Items for Systematic Reviews and Meta-Analyses) flow diagram of the literature review’s search process.

Of the 776 unique review articles screened, 88 (11.3%) related to DHIs for health care providers were included (Figure 1). Of these 88, 7 (8%) related to the identification and registration of persons [14-20], 19 (22%) to person-centered health records [14-16,18,21-35], 17 (19%) to health care provider decision support [14,19,21-23,25,30,31,36-44], 60 (68%) to telemedicine [19,26-28,30,36,39,40,42,45-95], 15 (17%) to health care provider communication [14,16,19,24,25,36,37,47,68,72,77,96-99], 4 (5%) to referral coordination [22,37,38,75], 4 (5%) to scheduling and activity planning for health care providers [14,25,28,95], 15 (17%) to health care provider training [18,25,40-42,57,59,96,100-106], 1 (1%) to prescription and medication management [15], 5 (6%) to laboratory and diagnostic imaging management [14,22,25,77,107], and 2 (2%) to health care provider financial transactions [108,109] (Table 1).

Overall, 63 reviews drew on evidence solely from LLMICs, while the remaining reviews referenced DHIs used in both LLMICs and high-income countries. Across all reviews, DHIs implemented in African countries were the most common, followed by countries in South and Southeast Asia, South and Central America, East Asia and the Pacific, the Middle East, and Europe. Over the study period (ie, 2010 to 2024), the number of reviews published steadily increased.

In the subsequent subsections, we present the review results grouped by the 11 categories of DHI identified for use by health care providers in the *CDISAH*.

**Table 1:** Reviews included in the umbrella review and the digital health interventions they focused on.

Studies			WHO^a^ *Classification of Digital Health Interventions, Services, and Applications in Health* categories for health care provider digital health interventions																				
			Identification and registration of persons		Person-centered health record		Health care provider decision support		Telemedicine		Health care provider communication		Referral coordination		Scheduling and activity planning for health care providers		Health care provider training		Prescription and medication management		Laboratory and diagnostic imaging management		Health care provider financial transactions
**2024**																							
	Bostan et al [35]			✓																			
	Dilhani et al [88]							✓															
	Flores Aniotz et al [89]							✓															
	Khosravi et al [90]							✓															
	Knop et al [95]							✓						✓									
	Lestari et al [91]							✓															
**2023**																							
	De and Pradhan [92]							✓															
	Gayesa et al [93]							✓															
	Eslami Jahromi and Ayatollahi [94]							✓															
	Jensen et al [105]															✓							
	Kim et al [79]							✓															
	Madonsela et al [80]							✓															
	Mishra et al [81]							✓															
	Tiwari et al [82]							✓															
**2022**																							
	Ionescu et al [41]					✓										✓							
	Mahmoud et al [83]							✓															
	Guillaume et al [106]															✓							
	Bossman et al [84]							✓															
	Owolabi et al [42]					✓		✓								✓							
	Rahman et al [85]							✓															
	Solomon et al [86]							✓															
	Tahir et al [87]							✓															
**2021**																							
	Agarwal et al [44]					✓																	
	Feroz et al [109]																					✓	
**2020**																							
	Aung et al [45]							✓															
	Gonçalves-Bradley et al [43]					✓																	
	Avidor et al [46]							✓															
	Eze et al [47]							✓		✓													
	Flores et al [48]							✓															
	Fu et al [49]							✓															
	Godinho et al [50]							✓															
	Harrison et al [33]			✓																			
	Hoxha et al [34]			✓																			
	Kermani et al [51]							✓															
	Lemma et al [32]			✓																			
	Leochico et al [52]							✓															
	Mahmood et al [31]			✓		✓																	
	Mao et al [54]							✓															
	Merchant et al [53]							✓															
	Odendaal et al [19]	✓				✓		✓		✓													
	Riplinger et al [20]	✓																					
**2019**																							
	Ames et al [55]							✓															
	Ayanore et al [18]	✓		✓												✓							
	Britton et al [107]																			✓			
	Huang et al [40]					✓		✓								✓							
	Huang et al [100]															✓							
	Knight et al [56]							✓															
	Kononowicz et al [57]							✓								✓							
	Kyaw et al [102]															✓							
	Mayberry et al [58]							✓															
	McHenry et al [59]							✓								✓							
	Mishra et al [60]							✓															
	Siddiquee et al [61]							✓															
	Wang et al [62]							✓															
	Aamir et al [103]															✓							
**2018**																							
	Acharibasam et al [39]					✓		✓															
	do Nascimento et al [17]	✓																					
	Gibson et al [108]																					✓	
	Gimbel et al [30]			✓		✓		✓															
	Kumar et al [29]			✓																			
	Long et al [96]									✓						✓							
	Marcolino et al [63]							✓															
	Orton et al [38]					✓						✓											
**2017**																							
	Feroz et al [16]	✓		✓						✓													
	Kim et al [28]			✓				✓						✓									
	Latulippe et al [64]							✓															
	Njoroge et al [65]							✓															
	Pandian et al [104]															✓							
	Sarfo et al [66]							✓															
**2016**																							
	Akhlaq et al [67]							✓															
	Amoakoh-Coleman et al [97]									✓													
	Colaci et al [98]									✓													
	Eze et al [68]							✓		✓													
	Gorski et al [69]							✓															
	Hassibian and Hassibian [27]			✓				✓															
	Jawhari et al [15]	✓		✓														✓					
	Lee et al [26]			✓				✓															
	Lewis et al [70]							✓															
	Sondaal et al [71]							✓															
	White et al [3]							✓		✓													
**2015**																							
	Agarwal et al [37]					✓				✓		✓											
	Devi et al [73]							✓															
	Khanal et al [74]							✓															
	Pasquel et al [75]							✓				✓											
**2014**																							
	Aranda-Jan et al [101]															✓							
	Bloomfield et al [76]							✓															
	Hall et al [25]			✓		✓				✓				✓		✓				✓			
	Hubert et al [77]							✓		✓										✓			
	Peiris et al [14]	✓		✓		✓				✓				✓						✓			
	Goel et al [24]			✓						✓													
	Källander et al [23]			✓		✓																	
	Oluoch et al [21]			✓		✓																	
	Saliba et al [36]					✓		✓		✓													
	Noordam et al [99]									✓													
	Blaya et al [22]			✓		✓						✓								✓			
	Rey-Moreno et al [78]							✓															

^a^WHO: World Health Organization.

### Identification and Registration of Persons

In total, 7 reviews related to identifying and registering persons in LLMICs were reviewed [14-20]. Authors reported various digital tool applications, typically handheld smart devices privately owned or supplied by a health program and digital electronic medical record (EMR) systems. These applications included registering patients at the point of first contact (eg, during their first consultation), recording the health care services provided, tracking upcoming appointments, generating care follow-up lists, and providing a mechanism for clients to contact health care providers proactively. Ubiquitous technologies, particularly at the primary health care level and in the field where access to networked computers is rare, enhanced these applications. For instance, in Timor-Leste, midwives used smartphones to register pregnant women, receive notifications for follow-ups, access lists of clients due to give birth, send batch SMS text messaging for health promotion or service delivery, and facilitate client consultations. Clients could also request consultations with their midwife over the phone via a free SMS text messaging service [19].

Jawhari et al [15] reviewed the benefits and challenges of EMRs in low-resource settings, focusing on paper-to-digital record transformation challenges, distraction caused by user interfaces, determinants of user acceptance, and hardware or software barriers to implementation. While the quality of the available literature was often questionable, with many papers based on case reports and personal experiences, commonly emphasized process improvements associated with EMR implementations included improved efficiency of time-consuming or error-prone tasks, such as identity management. Specific benefits noted were clinic encounter management, human resource use, reduced chart filing times, improved continuity of care, reduced data integrity issues, and improved accuracy of reports. EMRs were also said to benefit clinic operations by allowing easy access to data and, in some cases, autogenerating information, which was reported to potentially reduce the time required to complete reporting requirements for government, donors, and supporters [15].

However, it was noted that unique patient identifiers are rarely used in LLMICs, resulting in challenges in identifying and tracking patients as well as retrieving medical records during care. Riplinger et al [20] highlighted that the inability to track patients accurately raises concerns about safety and quality across the care continuum. Mistakes during the registration or record retrieval process, such as linking a patient to the wrong medical record, duplicate records, similar or multiple patient names, or unknown dates of birth (often used to call records in the absence of a unique identifier), inhibit client identification processes, and such mistakes must be addressed for digital identification, registration, and tracking systems to work effectively.

### Person-Centered Health Records

In total, 19 reviews related to EMRs [14-16,18,21-35]. Authors of these reviews reported that EMRs were associated with improved health system functionality, better health care worker and patient experiences, and better patient outcomes. Improvements that were attributable to person-centered health records included the ability to collect and transfer health care data to a central server in real or near real time, even from handheld smart devices, such as personal mobile phones [24,26,31], leading to faster data processing, greater efficiency, fewer errors [25], and improved data completeness [16,21]. EMRs also enabled quicker retrieval of records and facilitated access to clinical data during consultations. EMRs were also reported to enhance the level of detail in medical records and improve legibility compared to paper-based systems [15]. Hassibian and Hassibian [27] reported that in rural areas, the ability to retrieve patients’ health records from “the cloud” from any location facilitated in-person clinical evaluations and enhanced the sharing of patient information with colleagues located remotely.

Studies reported improved staff satisfaction directly attributable to the digitally enabled ability to review a patient’s history before consultation and update it afterward. This capability empowered health care providers to tailor treatment to be more person centered [22]. Patient satisfaction also increased because of digital interventions, which reduced wait times [21] and facilitated access to personal health information [23]. According to Peiris et al [14], these factors improved patient attendance at noncommunicable disease services. Other studies reported improvements in the effectiveness, efficiency, and quality of care [15,30] as well as improved demand for public health interventions [28].

However, the implementation of EMRs could be challenging. Issues related to limited funding, sustainability, organizational and management leadership, infrastructure, data privacy and protection, ownership, and medico-legal barriers in the transition from paper to digital medical record keeping, as well as system design issues, such as overcomplicated, cumbersome, and user-unfriendly medical data entry tools that added burden to busy frontline health providers, were all mentioned [15,29,34,35]. Bostan et al [35] emphasized that the tendency to focus on technology distracts from the underlying (and typically more challenging to define and address) socioenvironmental and organizational barriers and that this impacts the proliferation of EMRs in LLMIC.

Notably, Jawhari et al [15] reported that EMR implementation does not improve health care efficiency or effectiveness; instead, digital systems tend to bring dysfunctional processes into focus and, in the short term, can exacerbate poor workflows. Researchers have suggested that optimizing paper-based medical record processes is a prerequisite for implementing digital systems [15]. Craig et al [7] suggested using digital EMR-aligned paper-based data collection forms to help bridge workflows during the transition from paper to digital record keeping. Harrison et al [33] added that a paper-based workflow during the transition could reassure staff that their jobs will not be replaced or drastically changed after the adoption of a digital system.

### Health Care Provider Decision Support

Clinical decision support systems have received considerable attention as a potential solution to address the shortage of trained clinical personnel in LLMICs. We examined 17 reviews related to digitally enabled health care provider decision support [14,19,21-23,25,30,31,36-44], which included 3 Cochrane reviews [19,43,44]. We found that most digital decision support tools aimed to ensure health care providers complied with standards and guidelines for health service delivery by applying algorithms for screening and making clinical management decisions, including medication choice and timely referral. There was a general agreement but low certainty in the evidence that well-executed digital decision support tools have the potential to circumvent structural and systemic barriers faced by health care providers in delivering care, with evidence suggesting that the use of mobile phones for health care delivery is feasible irrespective of the health care providers’ education level or prior training [22,37,44]. Two illustrative examples are provided in the literature. First, Owolabi et al [42] observed that essential tools, such as telephone calls and 2-way texting, were effective interventions in reducing loss to follow-up among surgical patients after discharge and that the capability to share files digitally and video chatting were valuable interventions when physical assessments were necessary but not logistically feasible; and, second, Gimbel et al [30] highlighted that using digital decision support systems in screening and triaging patients is possible without substantial investment in training or loss of performance when compared with established tools typically administered in a clinical setting.

In their Cochrane review, Agarwal et al [44] reported that the evidence for the effect of digital decision support intervention on patients’ and clients’ health behaviors was mixed and that the intervention probably makes little or no difference to specific behaviors (eg, smoking rates among people at risk of cardiovascular disease) but probably increases other types of desired behavior, such as adherence to treatment. Findings related to the effect of intervention on patients’ health status and well-being were also mixed. The authors stated that digital decision support intervention may slightly improve patient or client acceptability and satisfaction. However, they found no solid evidence that the interventions enhanced the time between presentation with an illness and appropriate management, provider acceptability or satisfaction, resource use, or unintended outcomes [44]. Similar conclusions were shared by Gonçalves-Bradley et al [43] in a separate Cochrane review published in 2020, which explored the impact of mobile technologies on care management and communication between health care providers.

Peiris et al [14] and Hall et al [25] noted that the benefits of digital decision support tools likely vary across settings and that features of the interventions, together with the motivation of health care providers and consumers to use them, access to technology, and whether the tools were combined with other quality improvement strategies, are factors determining success. Hall et al [25] nicely synthesized a broader sentiment across the literature reviewed: while DHIs tend to be disease or program specific, those that seek to improve clinicians’ decision-making capacity have broader system-wide benefits. They cited evidence from a randomized crossover simulation study that found that mobile health (mHealth)–based guidelines reduced clinical error rates by 33% and improved protocol compliance by 30%.

Odendaal et al [19], in their 2020 Cochrane review, focused on health providers’ perceptions and experiences using mHealth technologies to deliver primary care services. The authors concluded that they had moderate to high confidence that mHealth interventions (including clinical decision support systems) changed how health care providers worked together, delivered care, and engaged with clients and communities. They noted that while some health workers found decision support software useful, others found it threatened their clinical skills. While most health workers viewed mHealth as an improvement over paper-based systems, some perceived it as adding to their workload [19]. Ionescu et al [41] stressed the need for “institutional anchoring,” or the process of embedding a concept, initiative, and practice within a stable and recognized institution to ensure its long-term sustainability, legitimacy, and effectiveness. Furthermore, they suggested that the development of local expertise is needed to ensure health authorities (ie, the end users) have ownership over the development of digital systems and the local capacity to manage them.

### Telemedicine

The use of telemedicine to address health system challenges was the most featured DHI in the literature reviewed, with 60 articles included [19,26-28,30,36,39,40,42,45-95]. The 2 most common health system challenges addressed through telemedicine were information sharing among health care workers and the effectiveness of care delivery. The use of telemedicine services was diverse and included delivering primary health care consultations from locations remote to patients; providing clinical advice during public health emergencies and trauma events; and supporting specialties such as teleradiology, telesurgery, teledentistry, telepathology, teleoncology, telerehabilitation, and palliative care, among others. A common sentiment was that telemedicine offers cost-saving opportunities for both health systems and consumers; helps overcome health care accessibility issues, particularly for those in rural or remote areas or those who, because of disability, find it difficult to access appropriate health services; supports adherence to treatment; increases demand for care; allows consumer-centered care; and is adaptable. In addition, reviews cited the efficacy of telemedicine in diagnosing diseases, preventing community disease transmission, servicing older adults or patients receiving palliative care, and minimizing the risk of health care–associated infections [17,20,34,82,83,88]. For example, Kim et al [79], who conducted a meta-analysis of randomized control trials exploring the effectiveness of telemedicine on mental health outcomes, found that digital health intervention groups had lower postintervention depression and anxiety symptoms than controls. Rahman et al [85] pooled evidence related to the impact of mHealth on pregnancy care and found that it improved antenatal care service use dramatically and—if combined with 2-way communication—also increased the use of skilled birth attendance during delivery. Gayesa et al [93] supported these findings, adding evidence for the positive impact of mHealth on clients’ knowledge of obstetric danger signs, postnatal care use, and exclusive breastfeeding.

Multiple studies linked the rise in telemedicine adoption to the advancement of digital platforms and widespread smartphone ownership [27,30,61,67,80,94]. Tiwari et al [82] reported a >90% patient satisfaction with telemedicine services, which was supported by Madonsela et al [80], who highlighted positive perceptions among adolescents toward telehealth services, citing that these services helped overcome barriers, such as cost, anonymity, access, and stigma. The literature reviewed commonly cited increased accessibility for patients, service use, and satisfaction as benefits of telehealth services. In contrast, unfamiliarity with technology, lack of support, concerns for confidentiality and data security, personal data costs, limited internet access, and a general lack of trust in technology were identified as barriers. The authors warned that if these barriers are not addressed, they may contribute to unequal uptake, limited opportunity, and inequitable distribution of the benefits offered by telemedicine [19,27,40,53,55,80,82,88,92,94].

Tiwari et al [82] and Mahmoud et al [83] noted that community members and health care providers had developed a greater appreciation and acceptance of telemedicine services through increased exposure and experience during the COVID-19 pandemic. They concluded that the most significant barriers to the broader adoption of telemedicine were inadequate infrastructure and a lack of sufficient regulatory frameworks [82,83].

While there is consensus across the literature that, when appropriately implemented, telemedicine offers an attractive tool to support health service delivery, concerns remain about the robustness and transferability of research results as well as the lack of evidence regarding the costs and cost savings that ought to be incurred or expected. There is also a lack of evidence regarding the challenges health authorities face in sustaining the long-term use of telemedicine, the quality of care provided remotely, and the long-term client satisfaction with telemedicine-based models of care [26,50,54,55,79,80,83].

### Health Care Provider Communication

The impact of digital technology on health care professionals’ interpersonal peer communication was featured in 15 articles [14,19,24,25,36,37,47,68,72,77,96-99].

A key focus was improving maternal health services through mHealth [16,19,98,99]. Nearly half of the studies concluded that mHealth enhanced antenatal and postnatal care by promoting demand and reducing delays in seeking and receiving services. Another prominent theme was the enhancement of coordination and connectivity among health providers. In total, 4 articles found that digitally enabled communication between health care providers reduced delays in decision-making and improved the overall quality of care provided [36,72,77,99]. Odendaal et al [19] reported that health care professionals benefited from a greater ability to contact their colleagues, which helped foster collegiality and a collaborative environment for peer-to-peer knowledge exchange and learning. This was viewed as a novel way to provide professional and clinical supervision and support to health providers in rural and remote locations who would otherwise not have ready access to their professional networks.

Several challenges were noted, including hesitancy owing to personal privacy concerns, data security risks, fear of being monitored by employers, and worries that engaging with others through digital means would increase workload [19,24]. Moreover, 3 reviews highlighted technical issues, such as inadequate network coverage and unreliable power supply; these are common challenges faced in LLMICs that can undermine the stability and reliability of digital communication tools and reduce trust in these tools as a viable solution [19,24,72]. Finally, several authors highlighted that many studies have focused on pilot projects or small-scale implementations that tend to be descriptive. Consequently, there remains a lack of reliable, quality evidence on the long-term impact and effectiveness of digital health care provider communication interventions on primary care service delivery and outcomes [14,15,25,37,97,98].

### Referral Coordination

A total of 4 reviews described digital interventions that support referral coordination [22,37,38,75], ranging from those designed to support maternal and child health programs to noncommunicable diseases and dental care. Several articles described the improved effectiveness of digital referral systems over the standard. In Zambia, Orton et al [38] reported a marked improvement in patient referrals, including the ability to digitally schedule or communicate referral information and change details in near real time. Agarwal et al [37] reviewed 4 studies that ranged from less complex systems (eg, providing community-based health care providers with a mobile phone to contact a maternity ward when a pregnant woman requires care) to more sophisticated systems (eg, involving health worker–collected data feeding into algorithm-based automated alert systems).

Orton et al [38] also identified several challenges related to the utility and completeness of data in digital referral systems. These included a lack of detailed information collected in predesigned forms, which prevented accurate needs assessment by those receiving the referral, as well as binary data types that did not provide sufficient information to assess the impact of digital referral systems on improving service delivery. Further challenges included the limited software choices and a lack of standards to support digital referral integration with other systems, such as EMRs or appointment booking systems. In some cases, divergent approaches were implemented in the same country, resulting in unnecessary duplication and limiting opportunities for integration and scale-up. Furthermore, gaps related to how referral systems can engage patients throughout the entire continuum of care and inequitable access to mobile network coverage in rural and remote areas were highlighted. Ultimately, although there is enthusiasm surrounding the potential for digital health tools to support referral coordination, their impact remains inconclusive, particularly for use at scale, as evidence to date is mainly limited to health worker and client experiences.

### Scheduling and Activity Planning for Health Care Providers

In total, 4 reviews investigated digital tools for treatment scheduling and activity planning [14,25,28,95]. These included SMS text messaging–based patient reminders, software that supported health care providers in generating patient treatment schedules, and digital survey–based data capture and linkage to enhance health intelligence generation.

The most widely investigated applications were SMS text messaging–based patient reminders, which were consistently noted to improve patients’ compliance with appointment schedules. A child and maternal health program in Tanzania reported a significant reduction in the average number of overdue days for visitations from 9.7 to 1.4 days after the introduction of an SMS text messaging–based reminder system [25]. Similarly, Kim et al [28] and Knop et al [95] found that SMS text messaging reminders sent to women, when combined with equipping health care providers with digital planning and scheduling tools, augmented antenatal clinic attendance, improved vaccination coverage, and showed improvement in on-time vaccinations. However, some limitations were identified. These included time-consuming data transfers from mobile devices to web-based databases and the failure of SMS text messaging o reach some recipients, particularly in remote areas.

A digitized treatment scheduling initiative in Thailand, which used software to provide health care providers with follow-up schedules for patients with malaria, reported significant improvements in case follow-up rates, rising from 20% to 40% at baseline to >90% after the intervention [25]. In Haiti, a visual activity planning tool was developed that overlaid vaccination posts’ locations with population size and vaccine coverage. This created a visual interface that reportedly aided service delivery and tracking [28].

The authors noted that while digital tools to support scheduling offer opportunities to improve demand and make health systems in LLMICs more efficient, there is limited hard evidence on their effectiveness, as literature from LLMICs is drawn mainly from pilot studies that have rarely been followed up with more rigorous evaluations. In addition, interventions have generally not been scaled up and have yet to produce clear evidence of cost improvements or measurable impact on health outcomes.

### Health Care Provider Training

A total of 15 reviews discussed digitally enabled or facilitated health care provider training [18,25,40-42,57,59,96,100-106]. Broadly, positive impacts were seen to arise from using digital modes of health care provider training delivery in LLMICs, primarily by addressing barriers to access (ie, remote and asynchronous delivery) and the ability to reach more people with less budget (ie, achieving cost-effectiveness through economies of scale) [41,57,59,96,102,106]. SMS text messaging and mobile instant messaging were the most common methods for continuing education and online interaction between peers. For instance, Guillaume et al [106] found that across the 31 articles included in their review, participants generally had a high level of acceptability for using digital platforms for learning and interaction. They also reported that online peer interaction and mentorship contributed to positive learning outcomes. Furthermore, the authors reported that peer-to-peer interactions improved social support and reduced feelings of isolation.

Several challenges were identified in implementing and using digital technology for learning, including limited access to resources, such as internet coverage and stable electricity; scheduling flexibility for online participation; and familiarity with information technology among the targeted health care workers. Additional challenges included a poor understanding of effective online pedagogy, high upfront costs associated with developing curated education materials, the rigidity of predeveloped materials that hindered local adaptation, and a lack of technical support [40,41,57,101,103,106]. Owolabi et al [42] noted that remote education incorporating 2-way communication, such as telephone or videoconferencing, was more effective and should be integrated into digital training designs wherever feasible.

The authors concluded that while digital solutions for providing training opportunities in LLMICs offer great promise, further work is needed to build robust evidence and develop cost-effective and sustainable models that address the complex and diverse needs of the health workforce. They noted that current research primarily consists of evaluations using qualitative analysis focusing on the challenges faced in delivering digital training. They argued that to strengthen the evidence for the effective use of technology in addressing educational challenges in LLMICs, more research is needed on the phenomena and pedagogy of teaching and learning through digital modalities within these contexts [40,59,96,101,105].

### Prescription and Medication Management

Only 1 article systematically reviewed the impacts of EMRs on medication management in sub-Saharan Africa [15]. The authors analyzed 21 case reports and 11 observational studies, deducing that while the use of EMR in sub-Saharan Africa offers promise for improved medication management, the strength of the evidence was weak. The studies were descriptive, did not involve a comparison group, and could not measure the direct effect on health outcomes; instead, they tended to explore service delivery–related outcomes. Further research is required to identify the factors for successful implementation [15].

### Laboratory and Diagnostic Imaging Management

In total, 5 reviews investigated the use of digital health interventions by health care providers for laboratory and diagnostic imaging management [14,22,25,77,107]. Teleultrasound in LLMICs can “reliably produce satisfactory images with diagnostic utility that guide clinical management” [107]. Teleultrasound in LLMICs has been explored in the contexts of obstetric ultrasound and fetal echocardiography [107]; telecardiology [14,107]; telestroke [77]; teleopthamology for diabetic retinopathy screening; telesonography for developmental dysplasia of the hip, suspected mammary lesions, ovarian tumor, peritoneal tuberculosis, and renal abscess; and bedside point-of-care ultrasound scans performed by primary care physicians [107]. The ability to train nonexperts in remote areas to produce basic ultrasound imagery that can be reviewed by experts (both national and international) shows great promise for improving access to essential health services, improving patient outcomes, reducing referral times, and reducing costs associated with accessing diagnostic services [107].

Hall et al [25] evaluated a Ugandan study of teleimaging using sensors attached to mobile phones. The sensors had sufficiently high resolution to detect pathogens and hematological indications on microscope slides, enabling remote review for the diagnosis of malaria and tuberculosis. In Botswana, for example, a cervical screening study using mobile phone pictures sent via multimedia messaging service for remote evaluation reported reduced patient referral delays and travel times [25]. Similarly, the agreement between face-to-face consultation and remote image-based review by dermatologists to diagnose common skin diseases was found [25].

Regarding laboratory management, a web-based laboratory information management system in Peru was found to improve the quality of care of patients with tuberculosis, particularly by decreasing the time required for communicating results and improving the laboratory’s productivity [22]. The mChip (a blood-based HIV serodiagnostic test machine based on mobile phone technology with cloud-based medical records) was tested in Rwanda and demonstrated laboratory-level accuracy and real-time synchronization of patient health record data [25].

Frequently cited limitations in the reviewed articles for laboratory and diagnostic imaging management included small sample sizes, the absence of an appropriate control group, analysis by a single expert reviewer, and interobserver variability. These limitations preclude establishing the efficacy and impact of these interventions in addressing health system challenges. Furthermore, issues, including generalizability, mobile network coverage, and cost-effectiveness, remain unresolved, with studies cited as lacking methodological rigor (particularly randomized controlled trials) and with a limited focus on outcomes [14,22,25,26,75,77].

### Health Care Provider Financial Transactions

In the context of digital health, “health care provider financial transactions” refers to the digital processes and systems that facilitate financial interactions between health care providers and other entities, including patients, insurers, government agencies, and suppliers. This was one of the least well-addressed domains, with only 2 reviews [108,109] examining the use of digital technology to support health care provider financial transactions. These papers examined how digital financial tools, including online insurance claim portals, instant loans, and bank transfers, facilitate access to funds that enable patients to purchase health care services and products. Feroz et al [109] noted that the flexibility and anonymity of online banking might help overcome barriers to health care, including geographic isolation, social stigma, and language issues.

Gibson et al [108] synthesized the literature’s account of enablers of digital financial transactions, noting the importance of timing, frequency, and the modality by which money transfers occur in the efficacy and utility of these services. They build on this, highlighting the subsequent impact digitization within financial transactions could have on the health system, health-seeking behaviors, and clinical outcomes. The authors suggested that, in the future, digital analytics could be used to identify clients in need or at risk, allowing more intensive, personalized, and targeted incentives and messages to be delivered. The evidence supporting the cost-effectiveness of the digital approach compared to usual practice is lacking. The authors highlighted the sensitivities surrounding the impact on equity and disparities that the increased use and reliance on digital financial transaction tools and third-party providers may exacerbate.

## Discussion

### Principal Findings

We synthesized evidence from reviews to understand how DHIs are being used by health care providers in LLMICs to address health system challenges. Our analysis was framed using the WHO *CDISAH*, providing a structured lens through which to evaluate the scope and relevance of the existing evidence base.

Overall, the reviewed literature provides a general indication that DHIs are contributing to reshaping how health care providers in LLMICs interact with their clients, colleagues, and health systems. These transformations have the potential to address entrenched barriers to care delivery in underserved populations, supporting broader policy ambitions for UHC and the achievement of SDG 3. This underscores the importance for policy makers to consider DHIs not just as technological tools but as strategic enablers of equity and access in challenging service delivery contexts.

Although digital health is often promoted as a mechanism for improving resource efficiency, our findings highlight that this claim is not strongly substantiated in the context of LLMICs, particularly given the high transaction costs often associated with implementation. For policy makers, this signals the importance of context-specific cost-benefit assessments. Financial decisions around DHI adoption should be grounded in an understanding of the dynamic interplay between system needs, intervention design, available alternatives, and the long-term costs involved in sustaining DHIs. Importantly, assessments must account for both direct and indirect costs and benefits, including those that fall outside the health sector. To support sound investment and policy decisions, there is a pressing need for implementation science and health economics research to quantify the full value proposition of DHIs in LLMICs. This is especially important in settings where trade-offs between competing priorities must be carefully considered to avoid undermining the delivery of essential health services or placing an undue burden on health systems.

The literature refers to digital innovations that have been short lived or failed owing to misalignment between system design and contextual realities; differing priorities among end users, system designers, and funders; or an assumption that a “one size fits all” approach is appropriate across diverse contexts. These failures not only risk wasting limited resources but also undermine the generation of generalizable evidence on the impact, cost-effectiveness, and scalability of DHIs. Given the considerable investments required to establish the enterprise architecture (ie, the technology, policies, processes, and people) required for digital health to succeed, decision makers need robust evidence to justify the prioritization of DHIs over other critical system infrastructure, such as staff, medicines, and facilities. This reinforces the recommendation that operational research to evaluate DHIs must be integrated and budgeted as a core component of implementation. Furthermore, publication and open-access dissemination of findings are essential to ensure that learnings extend beyond local settings and inform regional and global digital health strategies.

Telemedicine emerged as the most frequently reviewed DHI, with 60 reviews cited. The literature identifies information sharing and enhanced delivery efficiency as key benefits, particularly in bridging barriers to care and stimulating demand. However, despite insights from the reviews, gaps remain in the evidence around quality of care, user experience, and long-term sustainability. These findings suggest that while telemedicine may hold considerable promise, its deployment must be supported by context-sensitive monitoring, evaluation, and learning frameworks. Policy makers and program managers should be cautious in scaling telemedicine solutions without evidence of their real-world performance, feasibility, and sustainability.

Several reviews note that health care providers’ widespread use of personal smartphones has facilitated the uptake of DHIs. Leveraging personal mobile devices may lower entry barriers and reduce system-wide costs for hardware, maintenance, and training. Moreover, familiarity and convenience likely enhance adoption. While privacy, cost shifting, and interoperability issues must be addressed, the literature supports mobile-optimized design as a pragmatic and user-aligned approach. For policy actors and implementers, ensuring DHIs are mobile friendly should be a core design principle, especially in settings where infrastructure constraints persist.

The observation by Jawhari et al [15] that digital systems often expose, rather than solve, systemic inefficiencies is pertinent. It underscores the importance of strengthening foundational health system building blocks, including workforce, financing, governance, and supply chains, alongside digital investments. For policy makers and international funders, this finding is a crucial reminder that DHIs should not be positioned as a shortcut to system reform but as a complement to broader, structural efforts to build resilient health systems.

Finally, common challenges to DHI adoption in LLMICs, such as dependency on external financing, limited local expertise, high initial costs, and uncertain returns, underscore the need for cautious, phased, and contextually grounded digital strategies. Policy makers must weigh the potential risks of investing in (sometimes) unproven DHIs against the benefits of investing in proven health system functions. With their broader perspective, access to technical expertise, and insights from global experience, development agencies and donors have a critical role to play in guiding responsible and context-sensitive digital transition. Institutions such as the WHO and regional entities such as the Asian eHealth Information Network are well positioned to provide guidance, advocate for country-led digital health strategies, and help align innovations with long-term development goals.

This study is not without limitations. First, as an umbrella review, we drew data from multiple reviews, which inherently synthesize research and may miss important nuances. Furthermore, it is unlikely that all research is captured in the reviews, and some may be captured in multiple reviews. While we were aware of this, we did not systematically exclude content drawn from the same primary research in our thematic analysis. Second, we acknowledge that not all insights are reported in the peer-reviewed literature, and, as such, potentially important insights may have been overlooked. We also recognize that some of the reviews we analyzed drew on data from countries across the economic spectrum and that this likely means our synopsis is not entirely focused on the use of DHIs in LLMIC contexts, as diverse as they are. To account for this, we present this study as a synopsis of the literature and do not suggest that it captures all that is known. Third, due to the nature of our study and the articles reviewed, findings tend to be general and underemphasize the importance of local context, local dynamics, and local champions in the operationalization of new initiatives, including digital health. Fourth, we were unable to assess the quality of research or the risk of bias in the included literature, meaning readers must rely on their judgment regarding the rigor of the articles on which this umbrella review is based. Despite these limitations, the review is the first (to the authors’ understanding) to synthesize knowledge across so many review articles and, as such, provides a consolidated point-in-time understanding of what has been published about DHI use by health care providers in LLMICs to address health system challenges. Together with the 3 other proposed umbrella reviews to be published in this series (1 related to the DHIs used by each of the following groups: “persons [consumers],” “health care managers,” and “data service managers”), this work provides a comprehensive overview of current knowledge in the field.

### Conclusions

This umbrella review synthesizes evidence from the literature on the use of digital technology by health care providers in LLMICs to overcome health system barriers. The findings indicate, albeit with a variable level of confidence, that DHIs are helping transform how health care is provided in LLMICs and are contributing to how countries meet their UHC- and SDG-related ambitions. The review found that most published research focuses on the application of technology for telemedicine, resulting in a coherent body of evidence in this area. Conversely, evidence supporting other DHIs is limited in volume and depth. This suggests that further research across the spectrum of interventions is required to support decision makers in LLMICs to make prudent choices about their countries’ digital futures and, by doing so, address uncertainties about the risks and opportunity costs that exist.

## Data Availability

Data sharing is not applicable to this article as no data sets were generated or analyzed during this study.

## Appendix

Multimedia Appendix 1 PRISMA (Preferred Reporting Items for Systematic Reviews and Meta-Analyses) checklist.

Multimedia Appendix 2 Literature search strategies.

## Abbreviations

CDISAH: Classification of Digital Health Interventions, Services, and Applications in Health
DHI: digital health innovation
EMR: electronic medical record
LLMICs: low- and lower-middle-income countries
mHealth: mobile health
SDG: Sustainable Development Goal
UHC: universal health coverage
WHO: World Health Organization
