# Supplementary material for: Use of Technology to Support Health Care Providers Delivering Care in Low- and Lower-Middle-Income Countries: Systematic Umbrella Review
Source: J Med Internet Res. 2025 Jun 18;27:e66288. doi: 10.2196/66288 (PMC12223456; doi:10.2196/66288)
Supplement: Multimedia Appendix 2 [file jmir_v27i1e66288_app2.pdf]

Supplementary File B: Search strategies

**Pubmed**	(((((((((((((developing countries[MeSH Terms]) OR (third world countries[MeSH Terms])) OR (low income population[MeSH Terms])) OR (under developed countries[MeSH Terms])) OR (less developed countries[MeSH Terms])) OR ("global south")) OR ("LMIC")) OR ("low-and-middle-income")) OR ("lower-middle income")) OR ("lower middle income")) OR ("low and middle income")) OR ("emerging economies"))<br>AND<br>((((((((((((telehealth[MeSH Terms]) OR (telemedicine[MeSH Terms])) OR (telenursing[MeSH Terms])) OR (health information technology[MeSH Terms])) OR (medical informatics[MeSH Terms])) OR (ehealth[MeSH Terms])) OR ("digital health")) OR ("e-health")) OR ("mhealth")) OR ("m-health")) OR (clinical informatics[MeSH Terms])) OR ("virtual care")))<br>AND<br>((((((((((((((((universal coverage[MeSH Terms]) OR (Universal Health Insurance[MeSH Terms])) OR (Universal health care[MeSH Terms])) OR ("universal health coverage")) OR ("UHC")) OR ("service delivery")) OR ("essential medicines")) OR ("financing")) OR ("governance")) OR ("quality")) OR ("safety")) OR ("access")) OR ("equity")) OR (workforce[MeSH Terms])) OR (leadership[MeSH Terms])) OR (accountability[MeSH Terms]))<br><br>Filters applied: Review, Systematic Review, Humans, English.
**SCOPUS**	TITLE-ABS-KEY (("developing country" OR "developing countries" OR "LMIC" OR "low-and-middle income" OR "low and middle income" OR "Lower middle income" OR "global south" OR "emerging economy" OR "emerging economies") AND ("telehealth" OR "telemedicine" OR "telenursing" OR "digital health" OR "ehealth" OR "e-health" OR "mhealth" OR "m-health" OR "health informatics" OR "clinical informatics" OR "health information systems" OR "health information technology") AND ("universal health coverage" OR "universal health care" OR "UHC" OR "service delivery" OR "essential medicines" OR "financing" OR "governance" OR "quality" OR "safety" OR "access" OR "equity" OR "workforce" OR "leadership" OR "accountability")) AND (LIMIT-TO (PUBYEAR , 2022) OR LIMIT-TO (PUBYEAR , 2021)) AND (LIMIT-TO (DOCTYPE , "re")) AND (LIMIT-TO (LANGUAGE , "English"))
**Web of Science**	(ALL=(("developing country" OR "developing countries" OR "LMIC" OR "low-and-middle income" OR "low and middle income" OR "Lower middle income" OR "global south" OR "emerging economy" OR "emerging economies") AND ("telehealth" OR "telemedicine" OR "telenursing" OR "digital health" OR "ehealth" OR "e-health" OR "mhealth" OR "m-health" OR "health informatics" OR "clinical informatics" OR "health information systems" OR "health information technology") AND ("universal health coverage" OR "universal health care" OR "UHC" OR "service delivery" OR "essential medicines" OR "financing" OR "governance" OR "quality" OR "safety" OR "access" OR "equity" OR "workforce" OR "leadership" OR "accountability")))<br>AND<br>**LANGUAGE:** (English)<br>AND<br>**DOCUMENT TYPES:** (Review)<br><br>Indexes=SCI-EXPANDED, SSCI, A&HCI, CPCI-S, CPCI-SSH, BKCI-S, BKCI-SSH, ESCI, CCR-EXPANDED,
**EMBASE**	1 universal health coverage.mp. or universal health insurance/ or universal health care/<br>2 service delivery.mp. or health care delivery/

3	essential drug/ or essential medicines.mp.
4	workforce/ or health workforce/ or workforce.mp.
5	health care financing/ or health financing.mp. or health insurance/
6	leadership/
7	governance.mp. or "organization and management"/
8	accountabilty.mp.
9	health care quality/
10	safety culture/ or safety/
11	health care access/
12	health equity/
13	1 or 2 or 3 or 4 or 5 or 6 or 7 or 8 or 9 or 10 or 11 or 12
14	telemedicine/ or medical informatics/ or digital health.mp. or electronic medical record/
15	telehealth/
16	ehealth.mp. or telehealth/
17	medical information system/
18	health information technology.mp.
19	mhealth.mp.
20	m-health.mp. [mp=title, abstract, heading word, drug trade name, original title, device manufacturer, drug manufacturer, device trade name, keyword, floating subheading word, candidate term word]
21	e-health.mp. [mp=title, abstract, heading word, drug trade name, original title, device manufacturer, drug manufacturer, device trade name, keyword, floating subheading word, candidate term word]
22	14 or 15 or 16 or 17 or 18 or 19 or 20 or 21
23	developing country/
24	middle income country/ or LMIC.mp. or lowest income group/
25	low income country/ or lower middle income countr*.mp.
26	low-and-middle income countr*.mp.
27	(low and middle income).mp. [mp=title, abstract, heading word, drug trade name, original title, device manufacturer, drug manufacturer, device trade name, keyword, floating subheading word, candidate term word]
28	23 or 24 or 25 or 26 or 27
29	13 and 22 and 28
30	limit 29 to (human and English language and "review")
